# Development of a Potential Penside Colorimetric LAMP Assay Using Neutral Red for Detection of African Swine Fever Virus

**DOI:** 10.3389/fmicb.2021.609821

**Published:** 2021-04-23

**Authors:** Yang Wang, Junfei Dai, Yongsheng Liu, Jifei Yang, Qian Hou, Yunwen Ou, Yaozhong Ding, Bing Ma, Haotai Chen, MiaoMiao Li, Yuefeng Sun, Haixue Zheng, Keshan Zhang, Ashenafi Kiros Wubshet, Alexei D. Zaberezhny, Taras I. Aliper, Kazimierz Tarasiuk, Zygmunt Pejsak, Zhijie Liu, Yongguang Zhang, Jie Zhang

**Affiliations:** ^1^State Key Laboratory of Veterinary Etiological Biology, Lanzhou Veterinary Research Institute, Chinese Academy of Agricultural Sciences, Lanzhou, China; ^2^Department of Basic and Diagnostic Sciences, College of Veterinary Sciences, Mekelle University, Mekelle, Ethiopia; ^3^Federal State Budgetary Institution, All-Russian Research and Technological Institute of Biological Industry (VNITIBP), Moscow, Russia; ^4^Federal State Budget Scientific Institution “Federal Scientific Center VIEV”, Moscow, Russia; ^5^University Center of Veterinary Medicine JU-AU, Krakow, Poland

**Keywords:** ASFV, visual LAMP, one-step, diagnostics, neutral red, penside, *Asfarviridae*

## Abstract

African swine fever (ASF) has caused huge economic losses to the swine industry worldwide. Since there is no commercial ASF vaccine available, an early diagnosis is extremely important to prevent and control the disease. In this study, ASF virus (ASFV) capsid protein-encoding gene (p72) was selected and used to design primers for establishing a one-step visual loop-mediated isothermal amplification (LAMP) assay with neutral red, a pH-sensitive dye, as the color shift indicator. Neutral red exhibited a sharp contrast of color change from faint orange (negative) to pink (positive) during LAMP for detection of ASFV. The designed primer set targeting highly conserved region of the p72 gene was highly specific to ASFV and showed no cross-reactivity with other swine viruses. The detection limit for the one-step visual LAMP developed was 10 copies/reaction based on the recombinant plasmid containing the p72 gene of ASFV. More importantly, the developed one-step visual LAMP showed high consistency with the results of the real-time polymerase chain reaction (qPCR) method recommended by World Organization for Animal Health (OIE). Furthermore, the results demonstrate that the colorimetric detection with this LAMP assay could be directly applied for the whole blood and serum samples without requiring genome extraction. Based on our results, the developed one-step visual LAMP assay is a promising penside diagnostic tool for development of early and cost-effective ASF monitoring program that would greatly contribute to the prevention and control of ASF.

## Introduction

African swine fever (ASF) is an acute and highly fatal infectious disease caused by ASF virus (ASFV). ASF was reported for the first time in 1921 in Kenya (Montgomery et al., 1921) and it has on several occasions, escaped out of continental Africa into other continents such as Europe, South America, and Asia (Detray, 1963; Blasco et al., 1989; Dixon et al., 2013; Zhou et al., 2018). The morbidity and mortality of ASFV in domestic pigs could be as high as 100%, and until now there is no effective vaccine against the disease (Galindo and Alonso, 2017). The disease has taken a tremendous toll on the swine industry in countries where epidemics occur and has a serious impact on the international trade of livestock products. Moreover, ASF is listed as a notifiable disease by World Organization for Animal Health (OIE).

ASFV is an enveloped virus with a double-stranded linear DNA. It is also the only member of the Asfivirus genus, belonging to the *Asfarviridae* family (Alonso et al., 2018). The ASFV viron has an icosahedral symmetry with a diameter of 175 to 215 nm (Breese and Deboer, 1966). Its genome length is between 170 and 190 kb consisting of 151 open reading frames (ORF) (Chapman et al., 2008; Villiers et al., 2010). It can encode 151 to 200 proteins among which p72 is one of the main structural proteins (García-Escudero et al., 1998). The viral protein p72 accounts for 1/3 of the total protein of the virus and its amino acid sequence is conserved. Thus, p72 gene is often used as a target gene for detection of ASFV (Luo et al., 2017). Variability of the nucleic acid sequence within the 478bp region at the C-terminal of p72 gene allows the distinction of ASFV into 24 genotypes (Galindo and Alonso, 2017).

Although efforts have been made to develop a vaccine against ASFV, no safe and efficacious vaccines are available yet. Therefore, the most effective means to control the disease relies on early and sensitive detection, followed by strict biosafety measures. The main methods for diagnosis of ASF include virus isolation (Gonzague et al., 2001), haemadsorption (Rowlands et al., 2009), antibody-based tests such as enzyme-linked immunosorbent assay (ELISA) (Hutchings and Ferris, 2006; Cubillos et al., 2013; Bergeron et al., 2017), indirect fluorescent antibody assay (IFA), immunochromatography test strip (ICTS) and molecular diagnosis (Notomi, 2000; Agüero et al., 2003; King et al., 2003; Basto et al., 2006; Giammarioli et al., 2008; Haines et al., 2013). Despite its good accuracy, virus isolation assay requires a good laboratory, well-trained personnel and is time consuming. On the other hand, diagnostic tests that detect antibodies may not be suitable for acute ASF cases, in where the infected animals succumb to the disease before generation of antibodies. Therefore, detection of ASFV mainly depends on molecular diagnostic assays. Among all the molecular diagnostic techniques, real-time polymerase chain reaction (qPCR) assay is one of the most widely used methods because of its high sensitivity, specificity, and rapidity, and is also one of the ASFV diagnostic methods recommended by OIE (OIE Terrestrial Manual, 2019). However, qPCR requires relatively expensive instruments and skilled professionals to operate. Moreover, the collected samples need be transported to the laboratory environment for qPCR testing, not only posing a biosafety risk but also delaying ASF confirmation. For such reasons, qPCR is not suitable for the penside test, especially in resource constrained settings. Therefore, it is imperative to develop more convenient and economical detection methods to enhance the overall prevention and control of ASFV in the world.

Loop-mediated isothermal amplification (LAMP) is a kind of nucleic acid amplification technique developed by Notomi (2000). This technique has the advantages of being highly specific, simple to operate, rapid reaction, good sensitivity, and low cost. In LAMP assay there is no time loss caused by temperature changes, mainly because the assay is carried out under isothermal condition of 60-65°C. So far, LAMP assays have been widely applied in the detection of human, plant, and animal pathogens, especially viruses, such as influenza virus, classical swine fever virus (CSFV), foot-and-mouth disease virus (FMDV) (Dukes et al., 2006; Imai et al., 2007; Chen et al., 2008, 2009). LAMP amplicons can be examined by agarose gel electrophoresis and ethidium bromide staining of the typical ladder-like pattern of DNA concatemers, yielded in DNA elongation during LAMP reaction (Notomi, 2000; Gill et al., 2008). The signal detection of LAMP can be achieved, either by applying specialized equipment, such as a real-time fluorimeter or real-time turbidimeter, or using naked-eye observation of color changes of the fluorescent intercalating dyes (e.g., ethidium bromide, SYBR green), or the complexometric calcein, a fluorescent metal indicator, under a UV lamp. For ASFV, p10 and p73 gene-based LAMP assays have been described (James et al., 2010; Wang et al., 2019). However, they have not been used for on-site testing mainly because the results were not read using the naked eye or the color changes were not obvious enough to easily differentiate between positive and negative reactions. To obtain a LAMP detection method in which the results could be visualized using the naked eye, addition of specific dyes in the reaction is desirable. There are mainly three dyes for visualization, hydroxy naphthol blue (HNB) (Goto et al., 2009), calcein (Tomita et al., 2008), and SYBR Green (Parida et al., 2005; Hill et al., 2008). These dyes are usually used separately; however, they share the same deficiency, that is the color differences between negative and positive reactions are not distinct enough. It is difficult to distinguish the results with weak color changes from the negative reaction, leading to the inaccurate interpretation of the results, especially when the sample concentration is too low. Given the demand of consumers, more chromogenic dyes need to be developed with great performance in LAMP reaction. Recently, Tanner et al. (2015) utilized the pH-sensitive indicator in a slightly buffered LAMP solution that generates color shift owing to hydrogen ions produced during DNA biosynthesis (Tanner et al., 2015). Neutral red chromogenic agent, which leads to color changes based on the pH value, can greatly improve the color change contrast of the LAMP positive and negative reactions, showing a great prospect of application.

In this study, a visual LAMP assay with neutral red based on p72 gene was successfully developed for early and rapid detection of ASFV in the field. The developed visual LAMP assay could accurately identify the nucleic acid as low as 10 copies per reaction and did not cross-react with other common swine viral pathogens. The undiluted serum and the EDTA blood could be directly detected without genome extraction, suggesting the potential use in the field, especially in resource-constrained settings. The developed assay provides a distinct negative and positive color difference by the discoloration performance of neutral red due to a pH drop caused by the hydrogen ions produced in the LAMP reaction. The detection results can be immediately identified by the naked-eye observation without the need of extra measurement equipment. We also compared the visual LAMP assay with qPCR recommended by OIE. Furthermore, this assay was evaluated with different genotypes of ASFV genomes and clinical samples without genome extraction.

## Materials and Methods

### Viruses and Tissue Samples

Classical swine fever virus (CSFV), highly pathogenic porcine reproductive and respiratory syndrome virus (HP-PRRSV), porcine circovirus type 2 (PCV2), porcine circovirus type 3 (PCV3), Japanese encephalitis virus (JEV), porcine parvovirus (PPV), porcine epidemic diarrhea virus (PEDV and pseudorabies virus (PRV) were used for specificity testing. In addition, 17 ASFV genomes of 5 different genotypes and 8 different types of clinical samples including EDTA blood, serum, spleen, kidney, liver, tonsil, lymph nodes and muscle tissue collected from domestic pigs were provided by Lanzhou Veterinary Research Institute, Chinese Academy of Agricultural Sciences (LVRI, CAAS). The details of the 126 samples are shown in Supplementary Table 1. All these samples were used both in the detection of ASFV with or without DNA extraction.

### Genome Extraction and Clinical Sample Handling

Complimentary DNA (cDNA) of CSFV, HP-PRRSV, JEV and PEDV were synthesized by reverse transcription kit (Promega, Madison, Wisconsin, United States) from total RNA extracted from infected cell culture supernatant. Genomic DNA of PPV, PCV2, PCV3, PRV, and tissues were purified using MiniBEST Viral RNA/DNA Extraction Kit Ver.5.0 (Takara, Dalian, China). All tissues were kept in liquid nitrogen before genome extraction. For field detection, 1-2 cubic centimeter of clinical specimens were collected using sterile tools and transferred to the sealed containers containing 5 ml phosphate buffer saline (PBS) and immediately hydrosealed with mineral oil to prevent cross contamination among samples. After collecting, tissue samples were homogenated with a handheld homogenizer and centrifuged at 3000 *g* for 5 min. An appropriate amount of supernatant under mineral oil was pipetted for detection.

### Primer Design

Primers were designed based on ASFV p72, which is highly conserved and frequently used for all kind of PCR detection of ASFV. Hence, a total of 524 p72 gene sequences were retrieved by BLASTN of ASFV-SY18-p72 (GenBank accession MH713612.1) sequence, of which only 60 had complete ORF (1941bp). Afterwards, 524 p72 gene sequences were aligned using CLUSTAL as implemented in MEGA7.0 software (Sudhir et al., 2016) to relatively find out the conserved regions. Five groups of LAMP primers for related conservative regions were designed by Primer5.0 as the candidate primers. All the primers were synthesized commercially (Tsingke Biotechnology Co., Ltd., Xian, China).

### Preparation of a Plasmid DNA Standard

The 1941bp fragment (GenBank accession No. MH713612.1) of the ASFV p72 gene was chemically synthesized and inserted into pUC57 plasmid (herein referred to as pUC57-p72) after E*co*RV (Tianyi Huiyuan Biotechnology Co., Ltd.) restriction digestion. The purified plasmid pUC57-p72 was serially 10-fold diluted, from 10^6^ to 10^0^ copies/μL, with nuclease-free water. The diluted pUC57-p72 were used as the template for sensitivity assay.

### OIE Real-Time PCR

For the real-time PCR, 2μL DNA template was added to a final reaction system of 25μL consisting of nuclease-free water (7.5μL), 2 × TaqMan^®^PCR mixture (12.5μL), 50pmol sense primers (1.0μL), 50pmol anti-sense primers (1.0μL) and 5pmol probes (1.0μL). The reaction system was amplified as previously described (King et al., 2003). *C*_*T*_-values were assigned for the samples using a threshold fluorescence value as previously described (King et al., 2003).

### Visual LAMP Assay With Neutral Red as an Indicator Dye

Visual LAMP assay with neutral red was performed in a total volume of 20μL which is consisted of 18μL LAMP reaction buffer (including 50mM KCl, 10mM (NH_4_)_2_SO_4_, 25μM neutral red, 8mM MgSO_4_, 0.1% Tween20, 0.5M betaine, 1.4mM dNTPs, 0.8 μM each of forward inner primer (FIP) and backward inner primer (BIP), 0.2μM each of forward loop (LF) and backward loop (LB) primers, 0.08μM each of forward outer (F3) and backward outer (B3) primers), 1μL 8.0U BST DNA polymerase (New England Biolabs Inc.) and 1μL DNA template with pH adjusted to∼8.5 with 1M KOH. The reaction system was kept below-20°C during storage and transportation until use. An appropriate amount of mineral oil was added to the mixture surface to prevent evaporation if the equipment has no hot-lid. In field condition (on penside), the visual LAMP reactions were incubated in a portable metal bath at 64°C for 45 min, and read the results directly by naked-eye observation. In the laboratory, the LAMP reactions were incubated in a qPCR amplifier (Bio-Rad, CFX96 Touch Real-Time PCR Detection System, Code No. CFX96) at 64°C for 45 min to collect the fluorescent signals. After the reaction finished, the time to positive (Tp) (Dukes et al., 2006) was defined when reactions reached a fluorescence threshold higher than the background fluorescence of ‘template-free’ control reactions. Additionally, the experimental results were directly discriminated the positive from others with the naked eye according to the color changes. Pink indicated a positive reaction while orange indicated a negative reaction. Furthermore, the results were also visualized by running on 1.2% agarose gel electrophoresis. The positive reaction showed a ladder-like pattern strip formation.

### The Restriction Endonuclease Digestion and Sequencing of Visual LAMP Products

The restriction endonuclease digestion was performed in a 20μL reaction system consisting of 2μL 10-fold M buffer (Takara, Code No. 1060A), 1μL *Hin*dIII (Takara, Code No. 1060A), and 5μg LAMP products. The reaction was incubated in a metal bath with a hot lid at 37°C for 60 min and visualized by running on 1.2% agarose gel electrophoresis. The Sanger sequencing was performed by Tsingke Biotechnology Co., Ltd., Xian, China.

### Comparison of Visual LAMP Assays With Different Indicator Dyes

The visual LAMP assays with neutral red, cresol red, HNB, calcein and SYBR green was performed separately as described below. All the visual LAMP reactions were incubated in a metal bath with a hot lid at 64 °C for 45 min, the detection results were defined by direct naked-eye observation under ambient condition or UV.

Visual LAMP assay with cresol red was performed in a 20μL reaction system which was the same as that of neutral red reaction mixture described above, in addition to replacing 1μL neutral red with 2.4μL cresol red.

Visual LAMP assay with HNB was performed in a 20μL reaction system consisting of 18μL LAMP reaction buffer (including 210μM HNB, 5mM MgSO4, 2μL 10 × buffer, 0.5M betaine, 1.6mM dNTP, 0.8μM each of forward internal primer (FIP) and backward internal primer (BIP), 0.2μM each of forward loop (LF) and backward loop (LB) primers, 0.08μM each of forward outer (F3) and backward outer (B3) primers), 1μL 8.0U BST DNA polymerase (New England Biolabs Inc.) and 1μL template DNA.

Visual LAMP assay with calcein was performed in a 20μL reaction system consisting of 18μL LAMP reaction buffer (including 1μL calcein solution providing manganese ion (Eiken China Co., LIT), 5mM MgSO4, 2μL 10 × buffer, 0.5M betaine, 1.6mM dNTP, 0.8 μM each of forward internal primer (FIP) and backward internal primer (BIP), 0.2μM each of forward loop (LF) and backward loop (LB) primers, 0.08μM each of forward outer (F3) and backward outer (B3) primers), 1μL 8.0U BST DNA polymerase (New England Biolabs Inc.) and 1μL template DNA.

Visual LAMP assay with SYBR green was performed in a 20μL reaction system which was identical with that of calcein reaction mixture described above, in addition to replacing 1μL calcein by 1μL 20 × SYBR green and the SYBR green dye was added to the system after the reaction was terminated.

### Statistical Analysis

GraphPad software Inc. Prism Version 8, US was used for data analysis and the generation of the graphs. For all comparative analysis, the qPCR was used as the gold standard. All reactions were repeated at least once and were generally carried out in triplicate.

## Results

### Optimization of LAMP Assay

**FIGURE 1:**
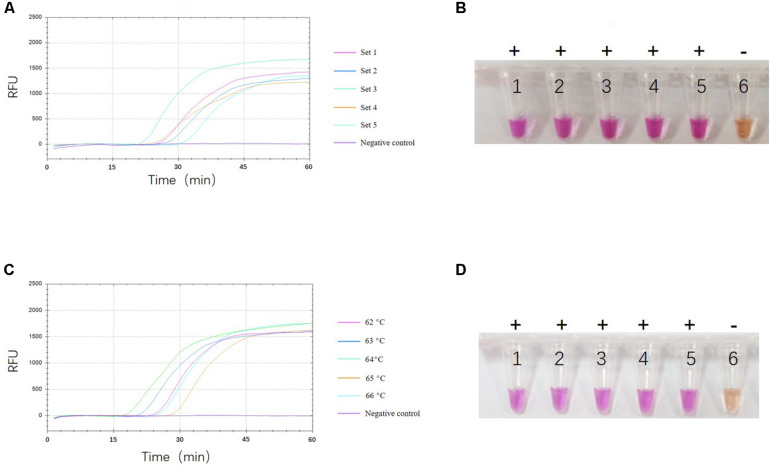
Selection of optimal reaction conditions. Analysis of optimal primer set **(A,B)** And reaction temperature **(C,D)** By fluorescence signals and naked eye observation. One representative result of three replicates is shown. **(A)** Shows the fluorescence signals of the five candidate primer sets. The fluorescence signals at each primer set are shown in a different color. **(B)** Shows the visual LAMP result of five candidate primer sets, numbers 1 to 6 represent primer set 1, set 2, set 3, set 4, set 5, and negative control, respectively. Positive reactions are shown by “+” and negative reactions are shown by “-”. **(C)** Shows the fluorescence signals of five different reaction temperatures. The fluorescence signals at each temperature are shown in a different color. **(D)** Shows the visual LAMP result of five different reaction temperature, number 1 to 6 represent 62°C, 63°C, 64°C, 65°C, 66°C and negative control, respectively. Positive reactions are shown by “+” and negative reactions are shown by “-”.

**FIGURE 2:**
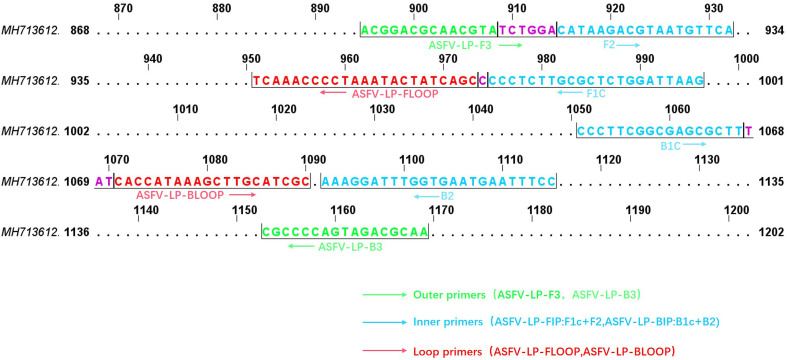
The target regions of p72 gene. Schematic showing location of primer recognition sites within p72 gene and the positions are numbered based on the complete p72 gene of ASFV-SY18 (GenBank accession MH713612.1). Primer sequences are shown in lower cases and are framed by a half box. Outer primers, internal primers (BIP and FIP primers are defined), loop primers are shown with green, blue, and red, respectively. The overlap region is shown with purple color.

To select the optimal visual LAMP primers, 524 sequences of ASFV p72 gene including 60 complete ORF were aligned to identify the highly conserved region for primer design. Five sets of primer candidates were selected and synthesized. The five different sets of primers were then evaluated with p72 gene at 10 copies/μL plasmid pUC57-p72. As showed in Figure 1A, the third primer set was the first generating fluorescent signals with a more uniform and brighter pink color while the negative control reaction turned orange (Figure 1B). Further evaluation showed that the third primer set was able to identify eight different 275 bp fragments in the highly conserved region of the p72 gene (Figure 2). Therefore, this primer set was used to establish a visual LAMP detection method for ASFV in this study. The primer sequences are shown in Table 1.

**TABLE 1:** Visual LAMP primers used for detection of ASFV based on the conserved region of the p72 gene.

**Primer**	**Type**	**Position^*a*^**	**Sequence (5′–3′)^*b*^**
ASFV-LP-F3	Forward outer	895-914	ACGGACGCAACGTATCTGGA
ASFV-LP-B3	Reverse outer	1153-1169	TTGCGTCTACTGGGGCG
ASFV-LP-FIP (F1c+TTTT+F2)	Forward inner	974-996, 909-933	CTTAATCCAGAGCGCAAGAGGGG**TTTT**TCTGGACATAAGACGTAATGTTCAT
ASFV-LP-BIP (B1c+TTTT+B2)	Reverse inner	1051-1070, 1092-1115	CCCTTCGGCGAGCGCTTTAT**TTTT**GGAAATTCATTCACCAAATCCTTT
ASFV-LP-FLOOP	Forward Loop	951-974	GGCTGATAGTATTTAGGGGTTTGA
ASFV-LP-BLOOP	Reverse Loop	1068-1090	TATCACCATAAAGCTTGCATCGC

*^*a*^Position numbers are based on the complete p72 gene of ASFV SY18 strain (GenBank No. MH713612.1).^*b*^Bold face indicates the TTTT spacer between F1c and F2 or B1c and B2.*

To optimize the reaction temperatures of the visual LAMP assay, the reaction mixtures were heated from 62 to 66°C at 1°C interval for 60 min, individually. As shown in Figures 1C,D, the reaction results among different temperatures were close to each other. Finally, we chose 64°C as the optimal reaction temperature.

To confirm the optimal reaction time of the visual LAMP assay, we compared and analyzed the fluorescence values during the whole course of the reaction and found that the fluorescence value reached the peak and tended to be constant after 45 min (Figures 1A,C). Considering the amplification efficiency and the total detection time, 45 min was considered to be the most suitable reaction time.

### Comparison of Visual LAMP Assays With Different Indicator Dyes

**FIGURE 3:**
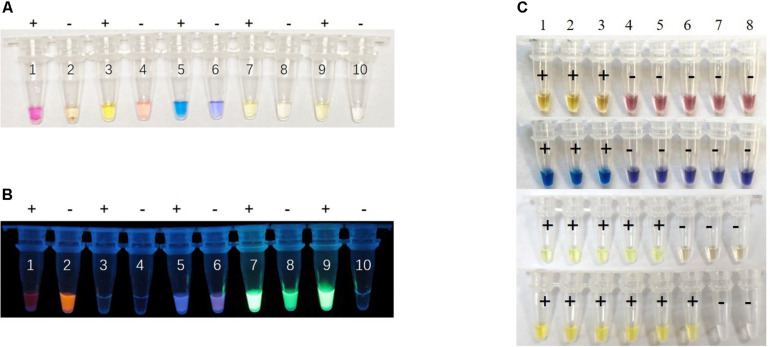
Performance comparison of different dyes for visual LAMP assay. **(A)** The visual LAMP results of different dyes by detecting 10^6^ copies/μL plasmid pUC57-p72 under daylight. Neutral red: number 1 to 2, cresol red: number 3 to 4, HNB: number 5 to 6, calcein: number 7 to 8, SYBR green 1: number 9 to 10. Positive reactions are shown by “+” and negative reactions are shown by “-”. **(B)** Visualization under UV irradiation. **(C)** The comparison of the sensitivity of different dyes by detecting pUC57-p72 standards, number 1 to 8 represent 10^6^ copies/μL, 10^5^ copies/μL, 10^4^ copies/μL, 10^3^ copies/μL, 10^2^ copies/μL, 10^1^ copies/μL, 10^0^ copies/μL, and negative control, respectively. The corresponding dyes from top to bottom are cresol red, HNB, calcein, and SYBR green. Positive reactions are shown by “+” and negative reactions are shown by “-”.

**FIGURE 4:**
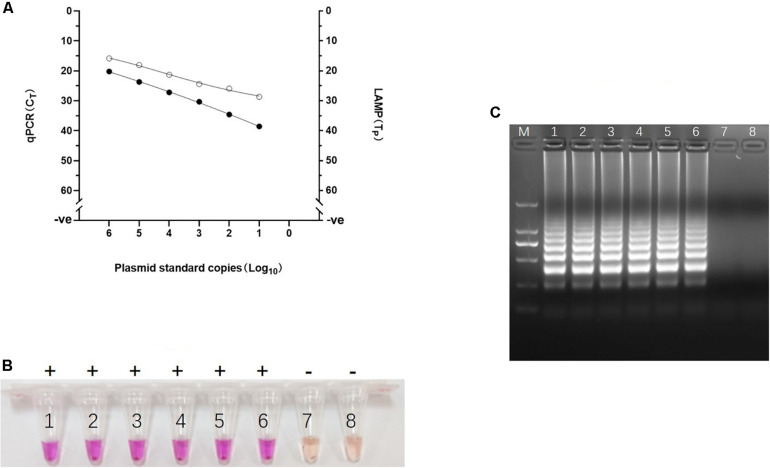
Analytical sensitivity of Visual LAMP assay. **(A)** Comparative analytical sensitivity of visual LAMP (open circles) and the gold standard qPCR (closed circles) using plasmid standards. **(B)** The visual LAMP results of the plasmid standards, number 1 to 8 represent 10^6^ copies/μL, 10^5^ copies/μL, 10^4^ copies/μL, 10^3^ copies/μL, 10^2^ copies/μL, 10^1^ copies/μL, 10^0^ copies/μL, and negative control, respectively. Positive reactions are shown by “+” and negative reactions are shown by “-”. **(C)** Determining the visual LAMP products by agarose gel electrophoresis. From left to right: lane M, DNA Marker DL-2000 (Takara); lanes 1-8, different copy number subjected to visual LAMP assay (10^6^ copies/μL, 10^5^ copies/μL, 10^4^ copies/μL, 10^3^ copies/μL, 10^2^ copies/μL, 10^1^ copies/μL, 10^0^ copies/μL and negative control, respectively).

To maximize the color discrimination, we also tested cresol red, HNB, calcein and SYBR green indicator dyes in the visual LAMP reaction by detecting pUC57-p72 (10^6^ copies/μL). We compared the color contrast between positive and negative reactions of different indicator dyes in visual LAMP under daylight and UV (Figures 3A,B). The results showed that the neutral red provided the sharpest color change (a shift from faint orange to pink) while cresol red (faint pink to yellow), HNB (violet to sky blue), calcein (yellow to yellow-green), and SYBR green (achromatic color to faint yellow) presented less significant color difference under daylight (Figure 3A). To additionally affirm the decision, we also ranked the different indicator dyes by conducting the performance tests of sensitivity and specificity. The results demonstrated that neutral red was better than the rest in the sensitivity (Figures 3C, 4B) while all dye markers could ensure the specificity (data not shown). Therefore, neutral red was superior compared to other dyes in the visual LAMP assay.

### Analytical Sensitivity and Specificity of Visual LAMP Assay

To evaluate the analytical sensitivity of the visual LAMP assay for ASFV, a series of dilutions of the recombinant plasmid pUC57-p72 containing p72 gene were prepared and then tested by visual LAMP assay. The results showed that the visual LAMP assay gave clear positive signal at 10 copies/reaction by either Tp analysis, direct naked-eye observation, or the agar gel-based electrophoresis. We further confirmed the specificity of the amplification by digesting the LAMP products by *Hin*dIII and sequencing (Supplementary Figure 1). The visual LAMP achieved equivalent analytical sensitivity to the gold standard qPCR (Figures 4A–C). In evaluating the visual LAMP specificity for ASFV, no cross reactions were observed with CSFV, HP-PRRSV, PCV2, PCV3, JEV, PPV, PEDV, and PRV while the positive control (the 10^6^ copies/μL pUC57-p72) were identified positively (data not shown), indicating that the visual LAMP assay was specific for detection of ASFV.

### Validation of the Visual LAMP Assay on Clinical Samples

**FIGURE 5:**
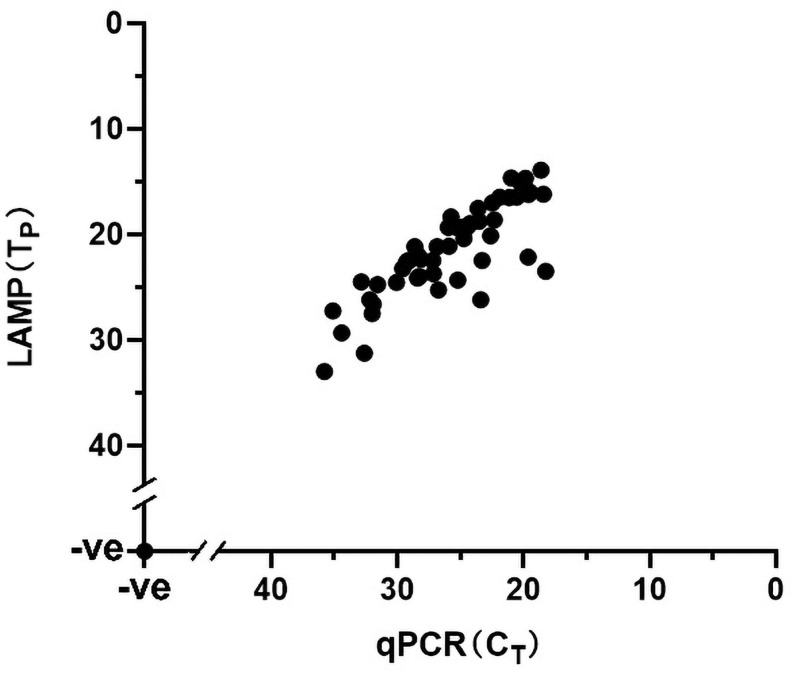
The detection results of clinical samples using Visual LAMP assay showing 100% concordance with the qPCR recommended by OIE. A total of 126 samples were used in this assay; the 52 positive samples (35 clinical samples and 17 different ASFV isolates genome) are shown in the graph whereas the 74 negative samples (66 clinical samples and nucleic acid of 8 different kinds of viruses) are represented by a single dot at the intercept of the *x*- and *y*-axis.

The performance of the visual LAMP assay was evaluated with clinical samples (n = 101). Of these 101 clinical samples tested (Supplementary Table 1), 35 samples were tested as positive for ASFV whereas the remaining 66 samples were negative by the visual LAMP. Based on a total of 101 samples examined indicating the diagnostic sensitivity and specificity of the visual LAMP assay for identification of ASFV was 100% and 100%, respectively, when compared to the qPCR recommended by OIE (Figure 5).

Additionally, all 17 ASFV isolates genomes were successfully amplified by the visual LAMP assay and then confirmed by qPCR whereas the cDNA from CSFV, HP-PRRSV, JEV, PEDV and DNA from PPV, PCV2, PCV3 and PRV tested negative (Figure 5). The data suggested that the visual LAMP assay was suitable for detecting different genotypes of ASFV.

### Evaluation of the Visual LAMP Assay Using Samples Without DNA Extraction

The process of DNA extraction is time-consuming and complicated, limiting application on penside. However, clinical samples usually contain various impurities, which may have negative influences on the DNA polymerization n. To explore the feasibility of the visual LAMP assay with the crude clinical samples, the experiments were carried out with eight most commonly collected clinical specimen for ASFV diagnosis including the-whole blood, serum, spleen lymph node, kidney, liver, tonsil, and muscle tissue, while 10^6^ copies/μL pUC57-p72, DNase/RNase-free water, cDNA or DNA from CSFV, HP-PRRSV, PCV2, PCV3, JEV, PPV, PEDV, and PRV were used as controls.

As expected, pUC57-p72 was tested as positive while RNase-free water and other viruses negative (data not shown). As summerized in Table 2, the crude EDTA blood and serum tested as positive yielding the same color changes (pink or orange) as that of the extracted DNA samples. However, not all other crude specimens could be identified as evenly as the subjects with DNA purification, suggesting the substances in the crude suspensions of the spleen, lymph node, kidney, liver, tonsil or muscle had negative impacts on the visual LAMP assay. The data indicated that the developed visual LAMP assay has its potential in a penside setting for ASFV detection, with the only 1μL of the crude EDTA blood or serum, which would greatly contributes to improve prevention and control of ASF, especially in the less-developed labs, pig farms, slaughter houses or even the ASF outbreak spots.

**TABLE 2:** Detection results of Visual LAMP assay using 8 different types of samples with or without DNA extraction.

**Sample details**	**Without DNA extraction**					**DNA extraction.**				
	**Sample No.**					**Sample No.**				
	**1**	**2**	**3**	**4**	**5**	**1**	**2**	**3**	**4**	**5**
Whole blood	+	+	+	−	−	+	+	+	−	−
Serum	+	+	+	−	−	+	+	+	−	−
Spleen	−	+	+	−	−	+	+	+	−	−
Lymph node	+	−	−	−	−	+	+	+	−	−
Kidney	+	−	−	−	−	+	+	−	−	−
Liver	−	+	−	−	−	+	+	+	−	−
Tonsil	−	−	−	−	−	+	+	−	−	−
Muscle	−	+	−	−	−	+	+	−	−	−

*Positive reactions are shown by “+” and negative reactions are shown by “−”.*

### Heterogeneity of Crude Sample pH in the Visual LAMP Assay

**FIGURE 6:**
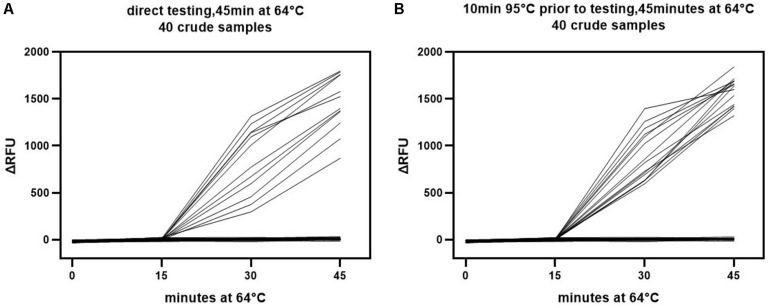
Colorimetric read-outs of visual LAMP assay over time for detecting crude samples. The colorimetric read-outs (ΔRFU) for the direct **(A)** And heat treatment **(B)** Crude samples were tested by visual LAMP assay. RFU was assessed every 15 min.

Comparison of the LAMP detection results for crude samples and isolated DNA revealed a much broader distribution of the △RFU value in crude samples (Figure 6A vs. Figure 6B). It was likely that the beginning pH variability of the crude samples impacted the detection results. This might have influenced the credibility of the assay. Subsequently, we explored whether this impact could be mitigated through simple treatment. The crude samples were subjected to a short heat treatment of 10 min at 95°C to denature the capsid and release the viral DNA as well as homogenize the sample. The outcomes showed that this improved the sensitivity and specificity of the visual LAMP assay (Table 3). We additionally found that the pH variability was positively associated with the sample volume utilized for LAMP assay. Consequently, an excess of the sample might adversely influence recognition. Overall, our results suggested better performance when utilizing heat treatment of crude samples prior to running the visual LAMP assay.

**TABLE 3:** Detection results of Visual LAMP assay using 8 different types of crude samples with or without preheating.

**Sample details**	**Without preheating**					**Preheating**				
	**Sample No.**					**Sample No.**				
	**1**	**2**	**3**	**4**	**5**	**1**	**2**	**3**	**4**	**5**
Whole blood	+	+	+	−	−	+	+	+	−	−
Serum	+	+	+	−	−	+	+	+	−	−
Spleen	−	+	+	−	−	−	+	+	−	−
Lymph node	+	−	−	−	−	+	−	−	+	−
Kidney	+	−	−	−	−	+	+	−	−	−
Liver	−	+	−	−	−	−	+	−	−	−
Tonsil	−	−	−	−	−	−	−	+	−	+
Muscle	−	+	−	−	−	−	+	−	−	−

*Positive samples are marked “+” and negative samples are marked “−”.*

## Discussion

In this study, a simple, rapid, specific, and sensitive ASFV detection method with its potential for penside testing was successfully developed based on the visual LAMP using neutral red, the pH-sensitive dye with sharp contrast, as the color change indicator for direct naked-eye observation under ambient condition. The strength of the developed visual LAMP derives from three main features: Colorimetric detection of LAMP reaction by using neutral red, the capability of detecting crude EDTA blood and serum as the target samples, and concise operation with almost no specific equipment and technological effort.

The first of these features offers easily discernable visualization. For the visualization of the LAMP result, the indicator dye is the key factor. There are mainly three kinds of dyes for current uses: the metal-sensitive indicators such as calcein (Tomita et al., 2008), hydroxy naphthol blue (Goto et al., 2009), and malachite green (Jothikumar et al., 2014; Nzelu et al., 2014), the intercalating dyes such as SYBR green (Parida et al., 2005; Hill et al., 2008) or pico green (Curtis et al., 2008; Wang et al., 2019), and pH-sensitive indicator dyes such as cresol red, neutral red, m-cresol purple and phenol red. In comparison with metal-sensitive and intercalating dyes, pH-sensitive colorimetric indicator offers two crucial advantages. Firstly, it provides sharp contrast for color changes, such as a shift from pale orange to pink (neutral red), red to yellow (phenol red, cresol red) or purple to yellow (m-cresol purple), while dark yellow to yellow (calcein) (Tomita et al., 2008), violet to sky blue (hydroxy naphthol blue) (Goto et al., 2009) and dark blue to light blue (malachite green) (Jothikumar et al., 2014; Nzelu et al., 2014). On the other hand, calcein requires manganese ion (Tomita et al., 2008; Arimatsu et al., 2012), which could inhibit polymerase reactions. Secondly, it reduces the risk of contamination to the minimal level by pre-addition of the dyes. Although intercalating nucleic acid dyes like SYBR Green can be put in the reaction system for visual detection, clear visualization is only available under UV (Goto et al., 2009). Additionally, a positive reaction is indicated by the color variation by adding an intercalating dye to the system after the reaction is completed. Such a colorimetric assay comes with an increased risk of contamination because the assay requires the opening of the reaction tubes. In our study, the pH-sensitive dyes including neutral red and cresol red showed more discernable color change advantages over HNB, calcein and SYBR green. In comparison of the difference of color changes between the positive and negative reaction tubes, neutral red was regarded as an outstanding colorimetric reagent for one-step ASFV visual LAMP assay by pre-addition of the dye.

The second feature is the capability of detecting ASFV in crude whole blood and serum without DNA extraction. Dynamic studies of ASFV shedding and excretion in pigs showed that ASFV was first isolated in blood among the inoculated pigs and higher ASFV titers were found in blood compared with other samples (Guinat et al., 2014; Mazur-Panasiuk et al., 2019; Zhao et al., 2019). Therefore, the whole blood and serum are the optimal materials for ASFV detection, either for ASF diagnosis in the outbreak or regular epidemiological survey.

The third feature is the concise operation offering two crucial advantages over qPCR. Unlike the qPCR, routine diagnosis technique for ASF, proper operation of this nucleic acid amplification assay offers the following advantages: (i) highly sensitive technique to detect ASFV DNA at a very early stage of the infection. And LAMP only needs simple instruments such as the water bath or metal block to guarantee stable temperature while qPCR requires specific amplification equipment such as precision thermocycling and the real-time fluorescent signals reader, (ii) The negative and positive results of the visual LAMP rely only on the direct observation of the color changes by naked eyes. It means the professionals are inessential to perform the LAMP assay while they are a prerequisite for the qPCR carryout.

Although two other visual LAMP assays were developed for detection of ASFV genome, they both were either more complicated in the reaction system components or less discernable color changes due to the indicator dye effects (James et al., 2010; Wang et al., 2019) in comparison with the assay developed in this study. James et al. (2010) reported that they used the novel lateral flow device to visualize dual-labeled biotin and fluorescein ASFV LAMP amplicons formed by conjugation of biotin and FITC at the 5′-terminus of FLoop and BLoop primers. In 2020, Wang et al. established the ASFV visual LAMP assay by adding 4-(2-pyridylazo) resorcinol monosodium salt, a kind of metal-sensitive indicator, under the existence of MnSO_4._ However, the color shift is from orange to light yellow, leading to being less easily discerned by naked eyes (Wang et al., 2019) when compared with the color change from pale orange to the pink of neutral red used in our visual LAMP for ASFV DNA identification. Furthermore, the results of our visual LAMP could also be detected by gel electrophoresis, characterized by the specific ladder-like bind formation, or by collecting the fluorescent signals in real-time using the Rox channel when ran in real-time PCR machine, showing the flexible alternative approaches for detection.

ASF diagnosis highly depend on the laboratory-based diagnostic techniques as ASF can be easily confused with several other diseases like pseudorabies (PR), classical swine fever (CSF), highly pathogenic porcine reproductive and respiratory syndrome (HP-PRRS). Our research showed that there were no cross-reactions between ASFV and CSFV, HP-PRRSV, PCV2, PCV3, JEV, PPV, PEDV, and PRV when they tested by the visual LAMP assay developed in our laboratory, which proved that this assay was an effective method to distinguish ASFV from other pathogens. Furthermore, the visual LAMP assay could be suitable for detecting almost all genotypes of ASFV, although only 5 genotypes (I, II, V, VIII, and IX) instead of 24 were verified in our current study, because the target region of p72 gene is highly conserved among all the genotypes. The visual LAMP was also proved to be as sensitive as the qPCR test recommended by OIE on both plasmid and clinical samples.

Additionally, the visual LAMP assay had also been evaluated whether it is suitable for the direct color discernable detection of ASFV DNA in the crude supernatant of the homogenized suspension of spleen, lymph node, kidney, liver, tonsil, and muscle tissue. To realize the wide use of this assay in the field, future validation is required with more different sample types, especially the oral or rectal swabs, blood powder in the fodder, the swill, water, or even the environmental samples. To fully assess the performance of the visual LAMP assay for on-site tests especially during the outbreak investigations.

The aerosol pollution frequently caused misreading of the results in LAMP detection is the main obstacle in its application, and it is also the most thorny problem encountered by numerous researchers. To solve this challenge, in laboratory settings, partition of the rooms or spaces according to functions, such as extraction DNA, constitution of reaction system, addition of DNA template, is a highly recommended approach, however, this is not suitable in field conditions. In this study, we used mineral oil as a sealant to seal on the liquid surface both in the reaction process and reaction sample collection, which could contribute to minimize aerosol volatilization to a certain extent. Meanwhile, for practical application, integrated design is also a key point. We need to build an integrated visual LAMP testing platform with rapid sample processing, fast LAMP amplification and accurate determination of results, so as to realize the systematization, portability, scientization and technicalization of LAMP diagnostic method. To achieve this, a lot of work, such as the methods of sample treatment and anti-contamination, the improvement of dyes and portable instruments, etc., still need to be further explored. In our research, to simplify the sample pretreatment, we made attempts to detect clinical samples without DNA extraction. However, except for the whole blood and serum, the detection effect of other types of samples is not ideal, which may be caused by the heterogeneity of specimen pH. Although we cannot completely exclude the negative effects caused by this factor for now, some simple pretreatment like heat treatment may alleviate this problem. In addition, in accordance with OIE Manual, more verification is needed to apply one diagnostic technique from the laboratory to the clinic. Therefore, there is still a lot of work to be done.

In general, the novel visual LAMP assay developed here is a specific, sensitive, rapid, simple-to-use, and cost-effective means for ASFV detection which can be used as filed and inside lab-diagnostic assay. The detection results can be observed by naked eyes immediately under ambient conditions when amplification is completed, presenting the sensitivity and specificity consistent with gold-standard qPCR assay. Consequently, a novel method that could be applied in the field and improve early warning systems of ASF was established in our study.

## Data Availability Statement

The raw data supporting the conclusions of this article will be made available by the authors, without undue reservation.

## Author Contributions

JZ, YZ, and ZL contributed to organizing and supervising the whole study and were also responsible for the funding acquisition. YW and JD conducted the experiments, analyzed the data, and drafted the manuscript. YL, HZ, YS, and KZ mainly contributed to designing the experiments and data analysis. QH, JY, YO, YD, BM, HC, ML, and AW mainly conducted the experiments and some data acquisition. AZ, TA, KT, and ZP analyzed the data and participated in the experiments designing and modification of the manuscript. All authors contributed to the article and approved the submitted version.

## Conflict of Interest

The authors declare that the research was conducted in the absence of any commercial or financial relationships that could be construed as a potential conflict of interest.

## Supplementary Material

Supplementary Figure 1 Results of restriction endonuclease digestion and sequencing of visual LAMP products. **(A)** Shows an agarose-gel visualization of LAMP product after *Hind*III digestion (lane2), the expected band size after restriction endonuclease digestion is 283bp. Undigested LAMP reaction product is also shown for comparison (lane1). Lane M, DNA Marker DL-2000 (Takara). **(B)** Shows the sequencing results of the enzyme digestion products in A. The alignment indicated the identity was 100% (263/263), with the exception of the 20 nucleotides flanking the two ends of the digested products mismatching the reference sequences causing by the sequencing error.
