# Supplementary figures and images for: Development of a Potential Penside Colorimetric LAMP Assay Using Neutral Red for Detection of African Swine Fever Virus
Source: Front Microbiol. 2021 Apr 23;12:609821. doi: 10.3389/fmicb.2021.609821 (PMC8102904; doi:10.3389/fmicb.2021.609821)

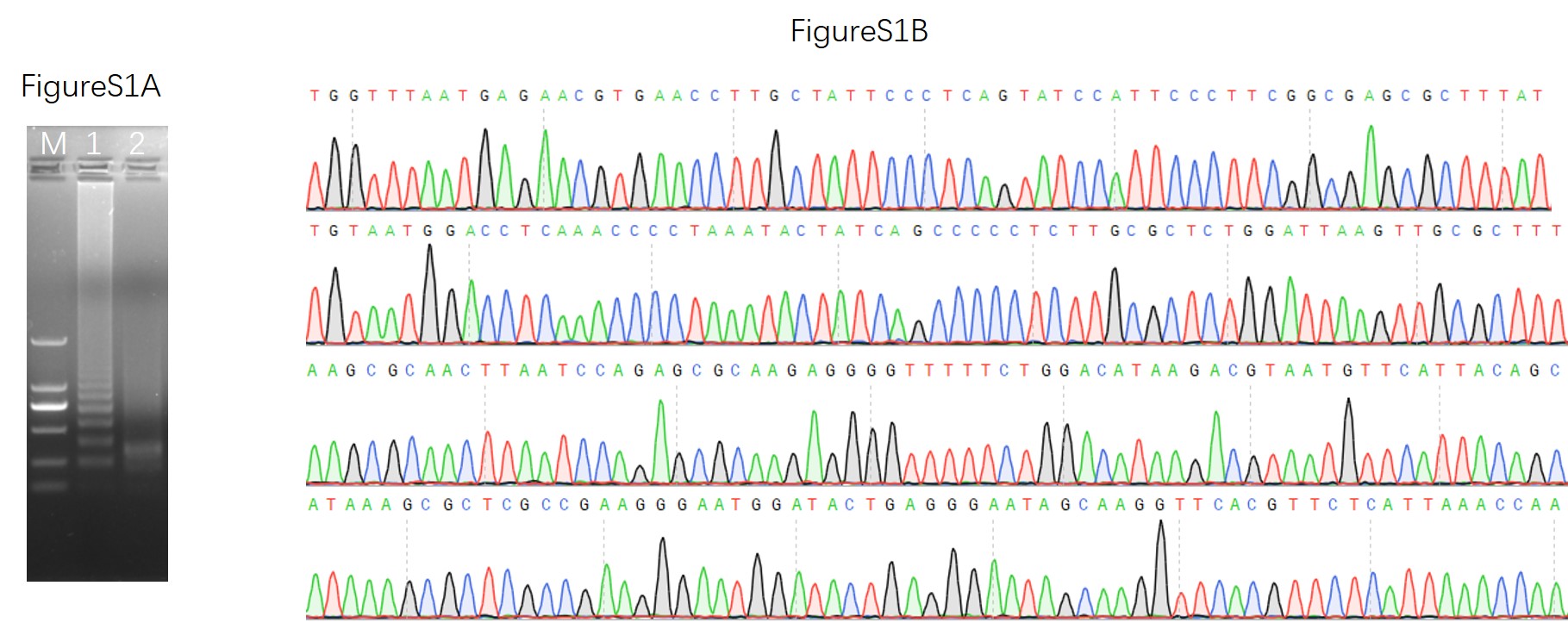

Supplement: Supplementary Figure 1 — Results of restriction endonuclease digestion and sequencing of visual LAMP products. (A) Shows an agarose-gel visualization of LAMP product after HindIII digestion (lane2), the expected band size after restriction endonuclease digestion is 283bp. Undigested LAMP reaction product is also shown for comparison (lane1). Lane M, DNA Marker DL-2000 (Takara). (B) Shows the sequencing results of the enzyme digestion products in A. The alignment indicated the identity was 100% (263/263), with the exception of the 20 nucleotides flanking the two ends of the digested products mismatching the reference sequences causing by the sequencing error. [file Image_1.JPEG]
