# Supplementary material for: Development of a Potential Penside Colorimetric LAMP Assay Using Neutral Red for Detection of African Swine Fever Virus
Source: Front Microbiol. 2021 Apr 23;12:609821. doi: 10.3389/fmicb.2021.609821 (PMC8102904; doi:10.3389/fmicb.2021.609821)
Supplement: Supplementary file 2 [file Table_1.DOCX]

**Table S1**

**Information of samples used in this study**

| No. | Details | genotypes | Sample type | qPCR Mean Ct |
| --- | --- | --- | --- | --- |
| 1 | CSFV | Un | cDNA | Un |
| 2 | HP-PRRSV | Un | cDNA | Un |
| 3 | JEV | Un | cDNA | Un |
| 4 | PEDV | Un | cDNA | Un |
| 5 | PPV | Un | Genome | Un |
| 6 | PCV2 | Un | Genome | Un |
| 7 | PCV3 | Un | Genome | Un |
| 8 | PRV | Un | Genome | Un |
| 9 | ASFV E70 | genotype I | Genome | 20.25 |
| 10 | ASFV Ba71V | genotype I | Genome | 29.57 |
| 11 | ASFV E75 | genotype I | Genome | 22.59 |
| 12 | ASFV Ss88 | genotype I | Genome | 24.57 |
| 13 | ASFV Lisbon60 | genotype I | Genome | 26.84 |
| 14 | ASFV Haiti | genotype I | Genome | 24.71 |
| 15 | ASFV Kat67 | genotype I | Genome | 32.83 |
| 16 | ASFV Ang72 | genotype I | Genome | 28.22 |
| 17 | ASFV Cv97 | genotype I | Genome | 22.30 |
| 18 | ASFV Nig01 | genotype I | Genome | 25.20 |
| 19 | ASFV BF07 | genotype I | Genome | 28.60 |
| 20 | ASFV Az08D | genotype II | Genome | 29.15 |
| 21 | ASFV Moz64 | genotype V | Genome | 27.19 |
| 22 | ASFV MwLil20/1 | genotype VIII | Genome | 30.05 |
| 23 | ASFV Ug03H.1 | genotype IX | Genome | 23.26 |
| 24 | ASFV Ken06.Bus | genotype IX | Genome | 25.75 |
| 25 | ASFV Ken08Tk.2/1 | genotype IX | Genome | 23.38 |
| 26 | EDTA blood | genotype II | Tissue | 28.35 |
| 27 | EDTA blood | genotype II | Tissue | 25.96 |
| 28 | EDTA blood | genotype II | Tissue | 18.23 |
| 29 | EDTA blood | genotype II | Tissue | 21.87 |
| 30 | EDTA blood | genotype II | Tissue | 26.73 |
| 31 | EDTA blood | genotype II | Tissue | 20.97 |
| 32 | EDTA blood | genotype II | Tissue | 24.43 |
| 33 | EDTA blood | genotype II | Tissue | 28.40 |
| 34 | EDTA blood | genotype II | Tissue | 23.59 |
| 35 | EDTA blood | genotype II | Tissue | 35.10 |
| 36 | EDTA blood | Un | Tissue | Un |
| 37 | EDTA blood | Un | Tissue | Un |
| 38 | EDTA blood | Un | Tissue | Un |
| 39 | EDTA blood | Un | Tissue | Un |
| 40 | EDTA blood | Un | Tissue | Un |
| 41 | EDTA blood | Un | Tissue | Un |
| 42 | EDTA blood | Un | Tissue | Un |
| 43 | EDTA blood | Un | Tissue | Un |
| 44 | EDTA blood | Un | Tissue | Un |
| 45 | EDTA blood | Un | Tissue | Un |
| 46 | EDTA blood | Un | Tissue | Un |
| 47 | EDTA blood | Un | Tissue | Un |
| 48 | EDTA blood | Un | Tissue | Un |
| 49 | EDTA blood | Un | Tissue | Un |
| 50 | EDTA blood | Un | Tissue | Un |
| 51 | EDTA blood | Un | Tissue | Un |
| 52 | Serum | genotype II | Tissue | 25.02 |
| 53 | Serum | genotype II | Tissue | 20.54 |
| 54 | Serum | genotype II | Tissue | 29.27 |
| 55 | Serum | genotype II | Tissue | 24.23 |
| 56 | Serum | genotype II | Tissue | 19.83 |
| 57 | Serum | genotype II | Tissue | 31.91 |
| 58 | Serum | Un | Tissue | Un |
| 59 | Serum | Un | Tissue | Un |
| 60 | Serum | Un | Tissue | Un |
| 61 | Serum | Un | Tissue | Un |
| 62 | Serum | Un | Tissue | Un |
| 63 | Serum | Un | Tissue | Un |
| 64 | Serum | Un | Tissue | Un |
| 65 | Spleen | genotype II | Tissue | 28.12 |
| 66 | Spleen | genotype II | Tissue | 18.42 |
| 67 | Spleen | genotype II | Tissue | 29.07 |
| 68 | Spleen | genotype II | Tissue | 22.46 |
| 69 | Spleen | genotype II | Tissue | 35.76 |
| 70 | Spleen | Un | Tissue | Un |
| 71 | Spleen | Un | Tissue | Un |
| 72 | Spleen | Un | Tissue | Un |
| 73 | Spleen | Un | Tissue | Un |
| 74 | Spleen | Un | Tissue | Un |
| 75 | Spleen | Un | Tissue | Un |
| 76 | Spleen | Un | Tissue | Un |
| 77 | Spleen | Un | Tissue | Un |
| 78 | Spleen | Un | Tissue | Un |
| 79 | Spleen | Un | Tissue | Un |
| 80 | Spleen | Un | Tissue | Un |
| 81 | Spleen | Un | Tissue | Un |
| 82 | Lymp node | genotype II | Tissue | 25.90 |
| 83 | Lymp node | genotype II | Tissue | 23.51 |
| 84 | Lymp node | genotype II | Tissue | 19.63 |
| 85 | Lymp node | genotype II | Tissue | 21.12 |
| 86 | Lymp node | genotype II | Tissue | 27.13 |
| 87 | Lymp node | genotype II | Tissue | 34.40 |
| 88 | Lymp node | Un | Tissue | Un |
| 89 | Lymp node | Un | Tissue | Un |
| 90 | Lymp node | Un | Tissue | Un |
| 91 | Lymp node | Un | Tissue | Un |
| 92 | Lymp node | Un | Tissue | Un |
| 93 | Lymp node | Un | Tissue | Un |
| 94 | Lymp node | Un | Tissue | Un |
| 95 | Lymp node | Un | Tissue | Un |
| 96 | Lymp node | Un | Tissue | Un |
| 97 | Lymp node | Un | Tissue | Un |
| 98 | Kidney | genotype II | Tissue | 19.60 |
| 99 | Kidney | Un | Tissue | Un |
| 100 | Kidney | Un | Tissue | Un |
| 101 | Kidney | Un | Tissue | Un |
| 102 | Kidney | Un | Tissue | Un |
| 103 | Kidney | Un | Tissue | Un |
| 104 | Kidney | genotype II | Tissue | 32.16 |
| 105 | Liver | genotype II | Tissue | 31.99 |
| 106 | Liver | genotype II | Tissue | 18.60 |
| 107 | Liver | Un | Tissue | Un |
| 108 | Liver | Un | Tissue | Un |
| 109 | Liver | Un | Tissue | Un |
| 110 | Tonsil | genotype II | Tissue | 31.54 |
| 111 | Tonsil | genotype II | Tissue | 25.32 |
| 112 | Tonsil | Un | Tissue | Un |
| 113 | Tonsil | Un | Tissue | Un |
| 114 | Tonsil | Un | Tissue | Un |
| 115 | Muscle | genotype II | Tissue | 32.59 |
| 116 | Muscle | genotype II | Tissue | 19.53 |
| 117 | Muscle | Un | Tissue | Un |
| 118 | Muscle | Un | Tissue | Un |
| 119 | Muscle | Un | Tissue | Un |
| 120 | Muscle | Un | Tissue | Un |
| 121 | Muscle | Un | Tissue | Un |
| 122 | Muscle | Un | Tissue | Un |
| 123 | Muscle | Un | Tissue | Un |
| 124 | Muscle | Un | Tissue | Un |
| 125 | Muscle | Un | Tissue | Un |
| 126 | Muscle | Un | Tissue | Un |
