# Supplementary material for: Development of a Potential Penside Colorimetric LAMP Assay Using Neutral Red for Detection of African Swine Fever Virus
Source: Front Microbiol. 2021 Apr 23;12:609821. doi: 10.3389/fmicb.2021.609821 (PMC8102904; doi:10.3389/fmicb.2021.609821)
Supplement: Supplementary file 3 [file Table_2.DOCX]

**Table S2.** The detection results of clinical samples using Visual LAMP assay showing 100% concordance with the qPCR recommended by OIE. A total of 126 samples were used in this assay；the 52 positive samples are composed of 35 clinical samples and 17 different ASFV isolates genome while the 74 negative samples are composed of 66 clinical samples and nucleic acid of 8 different kinds of viruses. “+” means positive samples while “−” indicates negative samples.

| Detection  Results | | OIE qPCR | |
| --- | --- | --- | --- |
|  |  | + | - |
| Visual LAMP | + | 52 | 0 |
|  | - | 0 | 74 |

Sensitivity=52/(52+0)=100%
